# Clinical Efficacy of Endoscopic Infratentorial Supracerebellar Approach for Pineal Region Tumors: A Retrospective Case-Control Study

**DOI:** 10.1155/2022/5702309

**Published:** 2022-08-10

**Authors:** Yaozu Zhu, Zhengwei Li, Zhongwei Xiong, Tingbao Zhang, Jincao Chen

**Affiliations:** ^1^Zhongnan Hospital of Wuhan University, Department of Neurosurgery, Wuhan, Hubei Province, China; ^2^The Affiliated Hospital of Hubei University of Arts and Sciences Xiangyang Central Hospital, Department of Neurosurgery, Xiangyang, Hubei Province, China

## Abstract

Tumors in the pineal region are deep, with complex surrounding anatomy, adjacent to important blood vessels and nerve structures, and surgical resection is difficult and risky. In this paper, we reviewed the literature to understand the epidemiology and clinical manifestations of pineal region tumors in the country and study the clinical indications, related problems, and successful experiences of patients with pineal region tumors treated by the transtentorial-superior approach. The clinical data of 80 patients with pineal region tumors were selected as the retrospective research objects and divided into the control group and the treatment group, with 40 cases in each group, according to the random number table method. The control group was treated using the endoscopic transtentorial approach (Poppen approach), while the treatment group was treated with the endoscopic supratentorial approach (Krause approach). The inflammatory factors, inflammatory stress response, postoperative neurological dysfunction, clinical efficacy, and poor prognosis were observed and compared between the two groups. Tumor resection and recurrence were used to compare the clinical outcomes of tumors in the pineal region. The extent of surgical resection was 100% higher in both groups, and the treatment group was comparable to the control group. The prognosis of patients after the operation was poor. Nausea and vomiting, visual disturbance, upper vision paralysis, and ataxia in the treatment group were significantly lower than those in the control group, with no statistical significance (*P* > 0.05). At the same time, the bone window can be reduced to reduce trauma and provide a certain reference for patients to choose a safe and complete resection method.

## 1. Introduction

The pineal region is located in the posterior part of the third ventricle, deep in the position, and the function of the surrounding structures is very important. It is bounded anteriorly by the third ventricle and the thalamus-occipital region, superiorly by the intermediate veil, the venous system of Galen, and corpus callosum, inferiorly by the apex of the midbrain and quadrigeminal, and it is surrounded by the transverse fissure, medial side of the occipital lobe, and the basal vein. Hence, tumor surgery in this site is quite difficult [1, 2]. However, the posterior boundary of the pineal gland is the space between the cistern and the cerebellum and the tentorium, which becomes the surgical channel for the infratentorial supracerebellar approach [3–5]. The most recent choice is the sitting position, which has the advantages of the natural sagging of the cerebellum to increase the size of the surgical channel because of gravity, good light introduction, natural bleeding out of the surgical field, relatively convenient operation for the operator, and low incidence of cerebellar swelling [6, 7]. Based on this, our hospital has carried out a certain exploration to explore the clinical efficacy of endoscopic infratentorial supracerebellar approach for tumors in the pineal region. The current research results are reported as given below.

In this paper, the clinical data of 80 patients with pineal region tumors were selected as the retrospective research object, and they were divided into the control group and the treatment group, with 40 cases in each group, according to the random number table method. The control group used the endoscopic transtentorial approach (Poppen approach), and the treatment group used the endoscopic supratentorial approach (Krause approach). The inflammatory factors, inflammatory stress response, postoperative neurological dysfunction, clinical efficacy, and poor prognosis were observed and compared between the two groups. The clinical outcomes of the pineal region tumors were compared using tumor resection and recurrence.

## 2. Material and Methods

### 2.1. Research Object

In this study, patients and their families have been informed, and they signed the informed consent. According to the calculation formula of the sample size of the cross-sectional survey, *n* *=* *t*_*a*_*2*PQ*/d*^*2*^*,n*, *n* is the sample size, *P* is the prevalence of tumors in the pineal region, *Q* = 1−*P*, and *d* is the allowable error. *a* = 0.05, *t*_*a*_ = 1.96. The minimum sample size brought into the formula was 80 cases, and the actual sample size included in this study was 80 cases. According to the random number table method, they were divided into the control group and the treatment group, 40 cases in each group. The clinical manifestations of tumors in the pineal region include increased intracranial pressure, Parinaud's sign, and the symptoms and signs of brainstem compression. With the development of imaging and laboratory technology, the preoperative evaluation of pineal region tumors has been greatly improved. Preoperative evaluation methods include head MRI, 3D-CT imaging technology, and serum markers of germ cell tumors. Detection and perfect imaging data can be used to observe the tumor from different angles before surgery to clarify the general shape of the tumor, the direction of growth, the source of the blood supply artery, and the relationship between the tumor and the deep cerebral venous system, which is the choice of surgical approach and intraoperative procedure. Complete tumor resection as much as possible to avoid damage to large blood vessels. The differences in general clinical data, such as age and body mass index, between the two groups had no effect on this study. For details, see Table 1.

### 2.2. Exclusion Criteria

Inclusion criteria are as follows:

- All patients in this study met the diagnostic criteria for pineal region tumors in the “imaging analysis of pineal region tumors” [8];
- All patients' diagnoses were confirmed by MRI examination in our hospital. There are no obvious contraindications to surgery and anesthesia before evaluating the heart, lung, liver, kidney, and other functions.
- Patients with tumor resection have complete clinical data and pathological imaging data as well as the clinical and imaging data of patients with shunt combined with gamma knife or radiotherapy.

Exclusion criteria are as follows:

- Patients with contraindications to surgery or with a history of drug allergy.
- Combined with other tumors of the central nervous system, nonpineal region tumors caused death or similar neurological dysfunction during follow-up, and follow-up data were incomplete.
- Smoked more than 10 cigarettes/day, alcoholics, poor compliance, influence judgment of effectiveness.

### 2.3. Nursing Intervention Methods

The control group was operated through the suboccipital tentorial approach (Poppen approach), i.e., the patient was in a sitting position, the neck was bent, the base of the horseshoe-shaped incision was toward the occipital region, the left edge of the bone flap was 1 cm away from the sagittal sinus, and the lower part was close to the upper nuchal line. The occipital lobe falls down because of gravity, and the occipital lobe can be retracted by pulling gently. The tentorium cerebellum is incised in parallel along the lateral 0.5–1 cm of the straight sinus, and the medial tentorial lobe is suspended and pulled in the direction of the falx. Sometimes, a wedge-shaped cerebellum can also be removed. Curtain flaps to increase exposure. The tumor and part of the deep vein in the pineal region were exposed, and the arterial supplying the tumor on the dura was cut off by electrocoagulation. The tumor was small and well-defined, and it could be completely removed after circular separation from the posterior pole of the tumor. When the tumor is large, intracapsular resection is performed first. After the tumor is shrunk, the boundary is carefully separated, and the tumor is completely removed. Pay attention to always face the back of the third ventricle when removing the tumor, and do not deviate to avoid injury to the thalamus.

In the treatment group, the surgery was performed through the infratentorial supracerebellar approach (Krause approach), i.e., the patient was in a sitting position, the head specimen was fixed on the tripod head frame, and the transverse sinus and sinus were located on the body surface through the superior nuchal line, external occipital carina, and other anatomical structures. Take the posterior occipital midline incision, about 7.0 cm long, 1/3 above the sinus confluence, and 2/3 below the sinus confluence. The subcutaneous tissue was carefully separated, and the attachment points of the posterior occipital muscles were separated on both sides along the squamous part of the occipital bone with a periosteal peeler to fully expose the occipital bone and locate the transverse sinus and sinus confluence. One bone hole was drilled under the sinus confluence, and a bone flap about 3.0 cm × 3.5 cm in size was milled with a milling cutter. After the bone flap was removed, the sinus confluence and bilateral transverse sinuses could be exposed. The dura was cut and sutured. The neuroendoscope was placed into the subtentorial space for observation, and the arachnoid membrane was carefully separated behind the quadrigeminal cistern to reveal the corresponding anatomical structure. Because the pineal region and its surroundings are deep, the exposure is often not ideal, and the cerebellum needs to be pulled down to fully expose it to sight. The exposure of the corpus callosum, pineal gland, superior and inferior colliculus, and their relationship with the surrounding structures were carefully observed under endoscopy. The tumor was excised in sections, and the tumor volume was reduced, and then the tumor cyst wall was carefully peeled off.

### 2.4. ObservationIndicators

1d and 7d after the operation, 3 ml of venous blood was collected from the elbow of the patient on an empty stomach in the morning, respectively, and the liquid chip method was used to detect malondialdehyde (MDA), superoxide dismutase (SOD), and glutathione peroxidase (CSH-px) levels.

### 2.5. Statistical Analysis

Use Epidata to enter all the data, and then use SPSS 25.0 to statistically process the data. The data needs to be entered into a computer database by a second person to ensure the completeness and accuracy of the data. *χ*^2^ test is used to express the count data as a percentage (%). For each parameter, data are mentioned as Mean ± SD and statistically analyzed by employing one-way ANOVA, followed by Tukey's multiple comparisons post hoc test. *P* < 0.05 is considered to be statistically significant.

## 3. Results

### 3.1. General Data Comparison

The gender, average age, average course of disease, average tumor volume, and other general data of the two groups of patients were compared by independent samples *t*-test, and there was no significant difference (*P* > 0.05). See Table 1.

### 3.2. Comparison of Inflammatory Stress Responses

**Figure 1:**
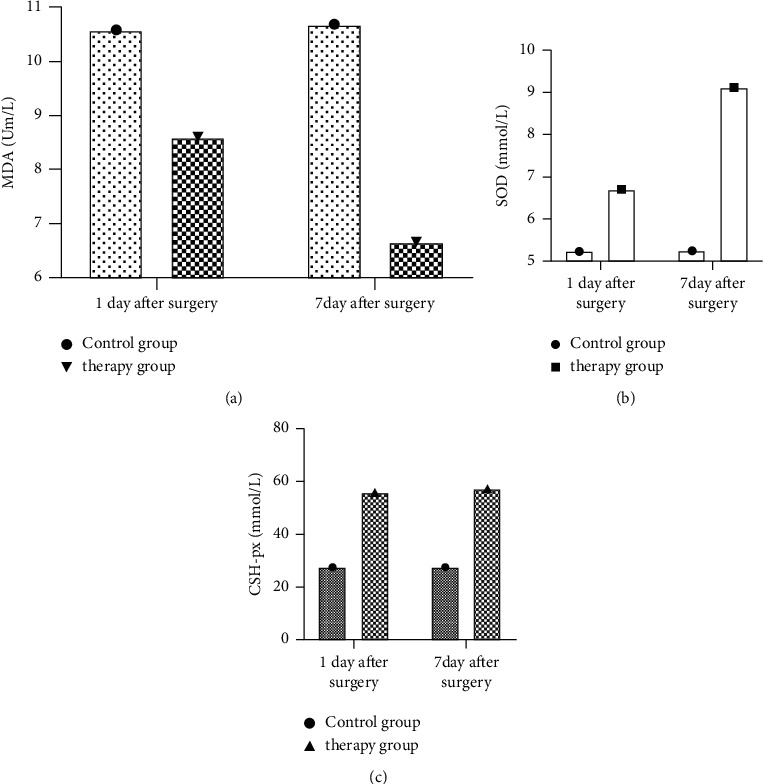
Comparison of inflammatory stress responses (after 1 day of operation, there was no significant difference in the inflammatory stress-related indicators between the two groups. After 7 days of operation, the MDA, SOD, and CSH-px of the two groups were significantly improved, and the treatment group was significantly better than the control group. They are mentioned as Mean ± SD and analyzed by employing one-way ANOVA, followed by Tukey's multiple comparisons post hoc test. The MDA, SOD, and CSH-px of the two groups were significantly improved, and the treatment group was significantly better than the control group (*P* < 0.001).

After 1 day of operation, there was no significant difference in the inflammatory stress response between the two groups (*P* > 0.05). After 7 days of operation, the MDA, SOD, and CSH-px of the two groups were significantly improved, and the treatment group was significantly better than that of the treatment group. In the control group, the above statistical comparison was significant (*P* < 0.05), as shown in Figure 1.

### 3.3. Comparison of Inflammatory Stress Responses

After 1 day of operation, there was no significant difference in the inflammatory stress response between the two groups (*P* > 0.05). After 7 days of operation, the MDA, SOD, and CSH-px of the two groups were significantly improved, and the treatment group was significantly better than that of the treatment group. In the control group, the above statistical comparison was significant (*P* < 0.05). See picture 1.

### 3.4. Poor Prognosis Comparison

**Figure 2:**
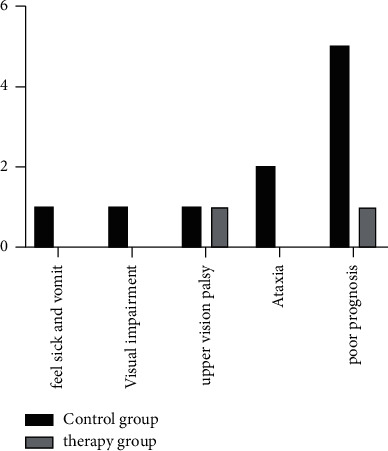
Comparison of the poor prognosis of postoperative patients showed that nausea and vomiting, visual disturbance, upper vision paralysis, and ataxia in the treatment group were significantly lower than those in the control group. Values are expressed as integers, and by chi-square test, it was found that nausea and vomiting, visual impairment, upper vision paralysis, and ataxia in the treatment group were significantly lower than those in the control group ((*P*) < 0.001).

The clinical efficacy of tumors in the pineal region was compared using tumor resection and recurrence. The surgical resection range of the two groups was 100% higher, and the treatment group was comparable to the control group. The comparison of the poor prognosis of postoperative patients showed that nausea and vomiting, visual impairment, upper vision paralysis, and ataxia in the treatment group were significantly lower than those in the control group, with no statistical difference (*P* > 0.05). See Figure 2.

### 3.5. Typical Cases

**Figure 3:**
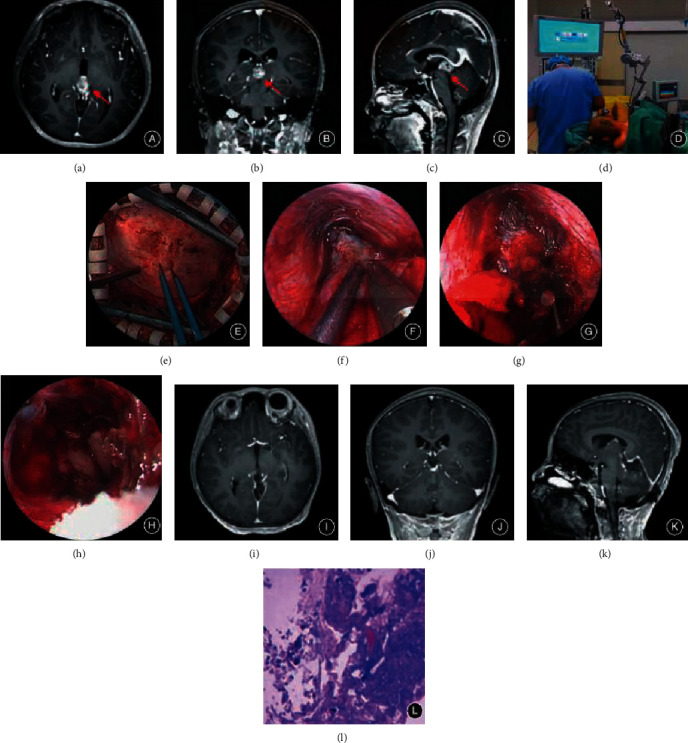
Clinical data of patients A–C. Preoperative head MR1 enhanced scans showed a circular space-occupying lesion in the pineal region in axial (a), coronal (b), and sagittal (c) views (arrows). (d) The patient's surgical position during the operation was supported by a pneumatic arm, the head of the operating bed was raised 15°, and the neck was flexed to the left by about 15°. (e) The transverse sinus was exposed during the operation. (f) The tumor was gradually revealed along the infratentorial and superior cerebellar vermis spaces, and the arachnoid membrane of the quadrigeminal cistern was explored. (g) Intracapsular decompression was performed to remove the tumor. (h) After the tumor was completely removed, the third ventricle was fully opened, and the opening of the midbrain aqueduct was seen to be unobstructed. MR scan of the skull showed that all the lesions in the pineal region were excised. (L) Postoperative pathological results suggested (pineal region) germ cell tumors (HE staining  ×  200).

The patient, male, was admitted to the hospital because of “headache”. Twelve days ago, the child had no obvious cause for headache, with paroxysmal pain, nausea, vomiting, and blurred vision. He was transferred to our department for further treatment after ventricular omaya extracapsular drainage in another hospital. Physical examination: consciousness, blurred vision in both eyes with double image, bilateral eyeball upward movement, cohesive movement disorder, and bilateral pupils are equal in size, about 3∗3 mm in diameter, and direct and indirect light reflexes disappear. The enhanced brain MRI in the other hospital suggested huge space-occupying lesions in the pineal region; obstructive hydrocephalus. After the operation, the child was transferred to the oncology department for radiotherapy, and the patient was admitted to the oncology department of our hospital by reviewing the head MRI 4 months after radiotherapy, considering the recurrence of the tumor. See Figure 3.

## 4. Discussion

Tumors in the pineal region are difficult to operate because of the complex adjacent relationship, important anatomical structure, and deep location. With the development of anesthesiology and imaging, especially the advent of CT and MRI, and the popularization of neurosurgery, direct surgical resection of lesions in this area has become possible, and the mortality rate has decreased significantly [9]. Therefore, the tumor at this site should be surgically removed as directly as possible, and precise neurosurgery and the selection of the best surgical approach are the keys to successful surgery [10]. At present, the representative surgical approach is the Krause transtentorial and supratentorial approach, and some authors use the combined approach of the supratentorial and supratentorial cerebellum. In this study, the Krause approach was adopted. We believe that the main features of this approach for the resection of tumors in the pineal region are as follows: the use of the natural gap between the cerebellum and the tentorium, the brain tissue is not damaged by surgery, the recovery after surgery is fast, and the complications are few. After the incision of the dura mater in the sitting position, the cerebellum naturally sags, and the gap is clearly exposed, and good surgical exposure can be achieved with little need for brain pressure plate traction [11]. The intraoperative anatomical position is clear, the lesion can be reached directly from the midline without further exploration, and the damage to the surrounding structures is minimized. The operation directly enters the third ventricle, the surrounding anatomical structure is clear, and the circulation of cerebrospinal fluid can be seen after the operation. Image navigation is used to further define the tumor boundary and surrounding important anatomical structures, especially to prevent damage to the central vein.

Ventricular drainage is usually performed preoperatively, or intraoperative puncture and drainage of the occipital angle of the lateral ventricle is performed to reduce the supratentorial pressure and prevent the herniation of the tentorial notch because of the large pressure difference between the upper and lower tentorium after the incision of the dura. Because of the long path of this approach, long microsurgical instruments are required [12]. When sitting, make the tentorium parallel to the ground. The elevation angle can easily cause air to enter the brain and cause tension pneumocephalus. The operation at the depression angle is difficult. When separating the tumor, pay attention to protecting the surrounding structures, especially the deep cerebral veins. Special attention should be paid to the dilation of deep veins. Reference [13]. When the tumor is large, because of the direct compression of the tumor, the internal cerebral vein and the great cerebral vein can be displaced. Pay attention to identification, and perform puncture if necessary to confirm that the circulation of the cerebrospinal fluid is restored after surgical resection. Intracranially, cotton pads can be used to gently pack [13]. After the operation, the wound surface was rinsed with warm saline, and the general requirements for seated surgery were paid attention to, including the bandage immobilization of the lower extremities, and slow changes in body position [14]. We summarize the key points of neuroendoscopic transinfratentorial supracerebellar approach for the resection of tumors in the pineal region, which is consistent with the traditional microscopic surgical approach. The implementation of neuroendoscopy via infratentorial supracerebellar approach also requires the adequate exposure of the transverse sinus and sinus confluence. If necessary, the sinus confluence and transverse sinus should be pulled upward to enlarge the surgical space [15]. The partial bridging veins of the superior vermis vein group can be selectively severed to obtain the best surgical field. However, the bridging veins in the superior cerebellar hemisphere vein group should be preserved as much as possible without affecting the surgical operation. The injury can cause cerebellar ischemia and edema. It should be fully evaluated before surgery, and the choice of bridging veins should be carefully considered [16]. Because of the occlusion of the superior cerebellar vermis and the influence of different inclinations of the tentorium, the lesions in the pineal region may be located below the surgical plane, and the cerebellar vermis can be pulled down during the operation and partially incised if necessary [17]. The posteromedial choroidal artery is the main source of blood supply to the pineal gland, and it is often pushed to one side in pineal tumors. When operating in the bilateral internal cerebral venous space during surgery, it should be avoided from behind the tentorial border and the vein of Galen.

We believe that as long as one can master the key points and precautions of seated surgery, equipped with a high-end surgical microscope, precision microsurgical instruments, and skilled microsurgery techniques, the subcanopy and supracerebellar approaches in the seated position should be used as the pineal region. Our experience is that the cerebellum naturally sags during the operation in the sitting position, and the cerebellum group can be well-exposed by being stretched, and the 2–3 cm gap can be reached only by the natural sagging of the cerebellum, which is enough for microsurgery to remove the tumor [19]. This approach is just aimed at the center of the lesion, and the relationship between the tumor and surrounding tissues can be clearly seen under a microscope, which can reduce damage to normal tissues. The tumor is located under the deep vein, and after the meninges are suspended upward, the great cerebral vein, the internal cerebral vein, the sinus confluence, and the transverse sinus are all retracted upward to be well-protected [20]. In this study, a spherical sac located on the cerebellum was seen during the operation, which was confirmed by puncture as an enlarged large cerebral vein. After carefully protecting the vein with gelatin sponge and pulling the vein slightly upward, the tumor was visualized and mostly resected until the third ventricle can be identified, indicating that this approach can be used even if the tumor is located deep in the deep vein. The supratentorial approach does not involve parietal or occipital lobe-related disability in interstitial operations. In this study, the MDA, SOD, and CSH-px of the two groups were significantly improved after surgery, and the treatment group was significantly better than the control group. These results indicate that endoscopic transinfratentorial and supracerebellar Krause approach can improve the inflammatory response, regulate coagulation function, and reduce the level of inflammatory indexes in patients with pineal region tumors. The specific reasons are as follows: after the tumor resection of the patient's pineal region, brain cells will cause intracellular ion disturbances, which will induce inflammation and generate a large number of oxygen free radicals [21, 24]. At the same time, when the body is stimulated by ischemia and hypoxia, it will cause oxidative stress to produce a large number of oxygen free radicals, resulting in a decrease in the levels of SOD and CSH-px and an increase in the level of MDA.

There are certain limitations in this study: firstly, the patients selected in this study were all patients with tumors in the pineal region, and patients with tumors in other body regions were not analyzed and studied one by one, resulting in errors in the results. Secondly, because of material and financial constraints, the inclusion and exclusion from our hospital are subject to a certain degree of subjectivity and the number is small, the follow-up time is short, and the research results may not be universal. Finally, this study cannot avoid the inherent bias and defects of retrospective research. In addition, this study does not have a large amount of data, and all the data are from the same hospital. Hence, the conclusions are less powerful. Because of the lack of statistical comparison between several surgical approaches, our choice of surgical approach was mainly based on the surgeon's experience and the available literature. This series also represents the ongoing evolution of indications and treatment strategies across various tumor types, reflecting the lack of consensus on their management. Therefore, the implementation of further prospective randomized controlled trials based on surgical modality, treatment strategy, and long-term follow-up will be more helpful to guide clinicians in the treatment of these patients.

## 5. Conclusion

In conclusion, the endoscopic infratentorial supracerebellar approach has obvious advantages for patients with pineal region tumors. It preserves the function of the cranial nerve to a certain extent, reduces the traction on the brain tissue, and can significantly reduce the occurrence of venous air embolism or venous embolism. It enables one to get timely relief, and at the same time, the bone window can be reduced to reduce trauma. [12].

## Figures and Tables

**Table 1:** .Comparison of general data between the two groups [n, ($\bar{x}$ ± *s*)].

Group	Gender (men/women)	Average age (age)	Average disease duration (moon)	Mean tumor volume(cm3)
Control group (40)	27/13	36.63 ± 8.32	6.31 ± 1.67	13.17 ± 2.34
Treatment group (40)	28/12	36.62 ± 8.31	6.33 ± 1.25	13.37 ± 2.20
*χ*2/*t*	0.058	0.005	−0.062	−0.394
*P*	0.809	0.996	0.951	0.695

## Data Availability

The experimental data used to support the findings of this study are available from the corresponding author upon request.

## Additional Points

The implementation of a whole-process informationized health management model combined with cardiac rehabilitation intervention for elderly patients with coronary heart disease after PCI can improve the quality of life and exercise endurance, and at the same time, it can improve the patient's self-care ability.

## References

[1] Sadashiva N., Deora H., Arumalla K. (2021). Pineal parenchymal tumor of intermediate differentiation (pptid) and papillary tumor of pineal region (ptpr): a review. *Neurology India*.

[2] Morgenstern P. F., Souweidane M. M. (2013). Pineal region tumors: simultaneous endoscopic third ventriculostomy and tumor biopsy. *World Neurosurgery*.

[3] Damgacı L., Hayat B., Güreşçi S. (2020). Papillary tumor of the pineal region with Parinaud syndrome: a case report. *Current Medical Imaging Formerly Current Medical Imaging Reviews*.

[4] Frosch K. H., Korthaus A., Thiesen D., Frings J., Krause M. (2020). The concept of direct approach to lateral tibial plateau fractures and stepwise extension as needed. *European Journal of Trauma and Emergency Surgery*.

[5] Fussell E. F., Krause A. L., Van Gorder R. A. (2019). Hybrid approach to modeling spatial dynamics of systems with generalist predators. *Journal of Theoretical Biology*.

[6] Schmalz G., Li S., Burkhardt R. (2016). MicroRNAs as salivary markers for periodontal diseases: a new diagnostic approach?. *BioMed Research International*.

[7] Korthaus A., Ballhause T. M., Kolb J. P., Krause M., Frosch K. H., Hartel M. J. (2020). Extended approach to the lateral tibial plateau with central meniscal subluxation in fracture repair: feasibility and first clinical and radiographic results. *European Journal of Trauma and Emergency Surgery*.

[8] Fang L., Qi S., Qiu B. (2008). Imaging analysis of tumors in the pineal region. *Chinese Journal of Neurology*.

[9] Choque-Velasquez J., Colasanti R., Resendiz-Nieves J. (2018). Papillary tumor of the pineal region in children: presentation of a case and comprehensive literature review. *World Neurosurgery*.

[10] Mathkour M., Hanna J., Ibrahim N. (2021). Papillary tumor of the pineal region in pediatric populations: an additional case and systematic review of a rare tumor entity. *Clinical Neurology and Neurosurgery*.

[11] Panke-Kochinke B., Krause G., Klimann O. (2015). [The scientific discourse about dementia in Germany-first results of the exemplary exercise of an integrative methodological approach]. *Pflege*.

[12] Krause K. L., DeDeaux C., Jung E., Than K. D. (2019). Two-level reverse Bohlman transsoseous approach for treatment of symptomatic pseudarthrosis. *British Journal of Neurosurgery*.

[13] Conradi E., Krause F., Boldt J. (2018). Forgotten approaches to care: the human being as neighbour in the German-jewish tradition of the nineteenth century. 2017 july 20. *Care in Healthcare: Reflections on Theory and Practice [Internet]*.

[14] Higgins G. S., Krause M., McKenna W. G., Baumann M. (2016). Personalized radiation oncology: epidermal growth factor receptor and other receptor tyrosine kinase inhibitors. *Recent results in cancer research. Fortschritte der Krebsforschung. Progres dans les recherches sur le cancer*.

[15] Krause A., Sperlich E., Schmidt B. (2021). Matsuda-Heck arylation of itaconates: a versatile approach to heterocycles from a renewable resource. *Organic and Biomolecular Chemistry*.

[16] Dixit C. K., Kadimisetty K., Otieno B. A. (2016). Electrochemistry-based approaches to low cost, high sensitivity, automated, multiplexed protein immunoassays for cancer diagnostics. *Analyst*.

[17] Krause S., Le Roux X., Niklaus P. A. (2014). Trait-based approaches for understanding microbial biodiversity and ecosystem functioning. *Frontiers in Microbiology*.

[18] Mittnik A., Wang C. C., Svoboda J., Krause J. (2016). A molecular approach to the sexing of the triple burial at the upper paleolithic site of dolní věstonice. *PLoS One*.

[19] Krause M., Foks H., Gobis K. (2017). Pharmacological potential and synthetic approaches of imidazo[4,5-b]pyridine and imidazo[4,5-c]pyridine derivatives. *Molecules*.

[20] Krause M., Hümpfner-Hierl H., Völker L., Hierl T., Pausch N. C. (2017). A new approach to treat bone gaps after midfacial and zygomatic fractures with a collagen membrane. *Oral and Maxillofacial Surgery*.

[21] Wang L., Lei X., Wang X. (2022). Efficacy and safety of PD-1/PD-L1 inhibitor chemotherapy combined with lung cancer fang No. 1 in relapsed and refractory sclc: a retrospective observational study. *Computational and Mathematical Methods in Medicine*.

